# Indocyanine green fluorescence in endoscopic transsphenoidal resection of pituitary neuroendocrine tumors: a systematic review

**DOI:** 10.1007/s00701-025-06500-z

**Published:** 2025-03-28

**Authors:** Ida Olesrud, Ingeborg Janshaug Halvorsen, Marit Aarvaag Storaker, Ansgar Heck, Daniel Dahlberg, Markus K. H. Wiedmann

**Affiliations:** 1 Department of Neurosurgery, Oslo University Hospital, Oslo, Norway; 2 Faculty of Medicine, University of Oslo, Oslo, Norway; 3 Department of Endocrinology, Oslo University Hospital, Oslo, Norway

**Keywords:** Indocyanine green, Pituitary adenoma, Fluorescence guided endoscopy, Endoscopic endonasal surgery, PitNET

## Abstract

**Background:**

Over the last decade, endoscope integrated indocyanine green (E-ICG) fluorescence has been introduced in endoscopic skull base surgery. E-ICG seems to be a promising tool for intraoperative tissue differentiation, distinguishing pituitary neuroendocrine tumors (PitNET) from pituitary gland. More recent technical advancements have made E-ICG with simultaneous near-infrared/white-light overlay imaging available. E-ICG may improve intraoperative tumor identification, enabling more precise surgery and ultimately improved patient outcome. This systematic review evaluates the use of E-ICG for PitNET surgery.

**Methods:**

A systematic review was performed in accordance with PRISMA guidelines. PubMed, EMBASE, MedLine and Scopus databases were searched using different terms for “pituitary adenoma” combined with “Indocyanine green”. Data from relevant original papers were extracted and analyzed.

**Results:**

Fifteen studies were included in the final analysis. The studies employed different ICG administration and fluorescence assessment protocols. Endpoints and methodology were heterogenous. Study populations varied from one to 39 cases. A total of 193 patients underwent transsphenoidal endoscopic surgery with E-ICG for PitNET. ICG dosage varied from 5 mg to 25 mg/kg. Thirteen studies administered ICG intraoperatively. Eleven studies utilized first-generation endoscopes, requiring toggling between near infrared light fluorescence and white light. Second generation dual or overlay mode endoscopes were used in four studies. Tumor fluorescence was assessed in eleven studies (141 cases). Six studies utilized a quantitative method to assess ICG-fluorescence. Seven studies specifically reported surgical complications. No safety issues regarding ICG use were reported.

**Conclusions:**

The current literature is mainly based on small single center cohorts and case-studies, presenting a wide variety of approaches. Procedures and intraoperative assessment of fluorescence were mainly performed utilizing first-generation ICG endoscopes. There is lack of consensus in terms of ICG as an intraoperative tumor marker. Endoscopic ICG seems a promising tool for intraoperative real-time tissue differentiation, including vascular structures, tumor and pituitary gland.

**Supplementary Information:**

The online version contains supplementary material available at 10.1007/s00701-025-06500-z.

## Introduction

Pituitary adenomas, more recently referred to as pituitary neuroendocrine tumors (PitNET) represent about 17% of adult primary brain tumors and consist of a heterogenous group of tumors in terms of clinical presentation, size and complexity [29]. The vast majority are histopathological benign [24], causing symptoms due to compression, invasion of adjacent structures or hormonal hyper- or hyposecretion. Surgical treatment, obtaining gross total resection (GTR), is the only potentially curative treatment strategy for most subtypes of PitNET [10, 25]. GTR is particularly important in hormone secreting PitNET in order to achieve endocrinological remission. Surgical resection of PitNET is considered a safe procedure with low morbidity and mortality [4, 17, 26, 38].

Methods for obtaining surgical access to the pituitary and sellar region have been subject to major technical developments [4, 17]. Despite technical advances, residual tumor rates for non-functioning PitNET (NFPAs) after surgery range from 10–36% [23]. Remission rates after surgery for functioning PitNET (FPAs) vary from 50–90% [8, 26, 34, 39]. The reported incidence of postoperative newly developed hypopituitarism varies across studies. Some European centers report an incidence of 6 to 16%, depending on definition [39]. However, the incidence might be underreported and subject to confounding or bias as pointed out by a Danish meta-analysis on endocrine function after TSS for NFPA [30]. The meta-analysis found postoperative pituitary failure rates in at least one axis in 0 to 36.6% (across 21 studies) [30].

Different subtypes of PitNET exhibit a variable degree of invasiveness into surrounding tissues. This necessitates optimal intraoperative visualization and surgical planning, to obtain the best possible surgical results [25]. GTR-rates vary significantly, from 84% at the best pituitary centers in the US, to e.g. 39% in a Danish study [21, 28]. Improved intraoperative, real-time fluorescence visualization during endoscopic surgery, might provide a useful tool for obtaining better GTR rates.

Indocyanine green (ICG) is a water-soluble cyanine dye that exhibits fluorescent properties under near-infrared (NIR) light. An intravenous bolus administration of ICG can be used as a real-time intraoperative angiographic marker, and the method is known as a safe and feasible tool in cerebrovascular surgery [3, 31]. ICG is generally considered safe. However, anaphylactic reactions can occur even at low doses [18]. Outside the cerebrovascular field, ICG has been used for intraoperative visualization of various tissues in both hemispheric and skull-base tumor surgery [3, 19, 36]. During pituitary surgery, it is thought that ICG could act as a vascular marker, illustrating differences in microvascular architecture between pituitary tumor and healthy pituitary tissue [22, 32, 35], enabling intraoperative visualization of a gland-tumor interface.

The objective of this study was to review the current literature on the use of E-ICG during endoscopic surgery for PitNET, with emphasis on reported versatility and feasibility. We assessed dosage and administration regimens, as well as endoscopic devices, and reported adverse events related to the use of ICG. We further assessed the evaluation of intraoperative visualization of tumor versus normal pituitary gland.

## Materials and methods

This study was exempt from local review board approval. Three separate systematic literature searches were conducted to identify original studies on ICG during endoscopic transsphenoidal surgery (ETSS) for pituitary adenomas (hereafter referred to as PitNET). The search was performed and completed by three independent reviewers (IO, IH, MS).

Medline (1949-), EMBASE Classic (1947-), PubMed and Scopus databases were searched using different terms for “pituitary adenoma” and “indocyanine green” in combination, including MeSH terms and keywords. The PubMed search string for example included (*"pituitary neoplasms"[MeSH Terms] OR ("pituitary"[All Fields] AND "neoplasms"[All Fields]) OR "pituitary neoplasms"[All Fields] OR ("pituitary"[All Fields] AND "adenoma"[All Fields]) OR "pituitary adenoma"[All Fields]) AND ("indocyanine green"[MeSH Terms] OR ("indocyanine"[All Fields] AND "green"[All Fields]) OR "indocyanine green"[All Fields]).* Detailed search strings for the different databases are presented as supplementary material (Supplementary Table 1).

### Inclusion criteria

Studies were eligible for inclusion if they met the following criteria: Original studies, including case series and case reports, investigating the use of ICG fluorescence during endoscopic transsphenoidal surgery for pituitary neuroendocrine tumors. Studies must report on the methodology and feasibility of ICG fluorescence for distinguishing pituitary tumor from normal pituitary gland during ETSS. Studies must specify the ICG dosage, administration protocol, and endoscopic system used for fluorescence imaging. Publications must be available in English. Full-text access must be available for data extraction and analysis. Studies must include human patients undergoing endoscopic transsphenoidal pituitary surgery with ICG fluorescence assessment.

### Exclusion criteria

Studies were excluded if they met any of the following criteria: Conference abstracts, book chapters, and review articles without original case series or case reports. Studies focusing on ex vivo, in vitro, or animal models. Studies that used ICG solely for intraoperative angiography (e.g., vascular visualization without assessing tumor and/or pituitary gland fluorescence). Studies where pituitary tumor and/or gland fluorescence assessment was not a primary or secondary endpoint. Studies with incomplete or ambiguous methodology that prevented meaningful data extraction.

Relevant literature was screened for technical aspects and ICG methodology, including dosage and timing, safety and adverse events, feasibility and efficacy in differentiating between tumor and pituitary gland. Data extraction and study analysis was based on the search results of February 6th, 2024. A final literature search was performed on December 12th, 2024, to update for recent publications.

**Fig. 1:**
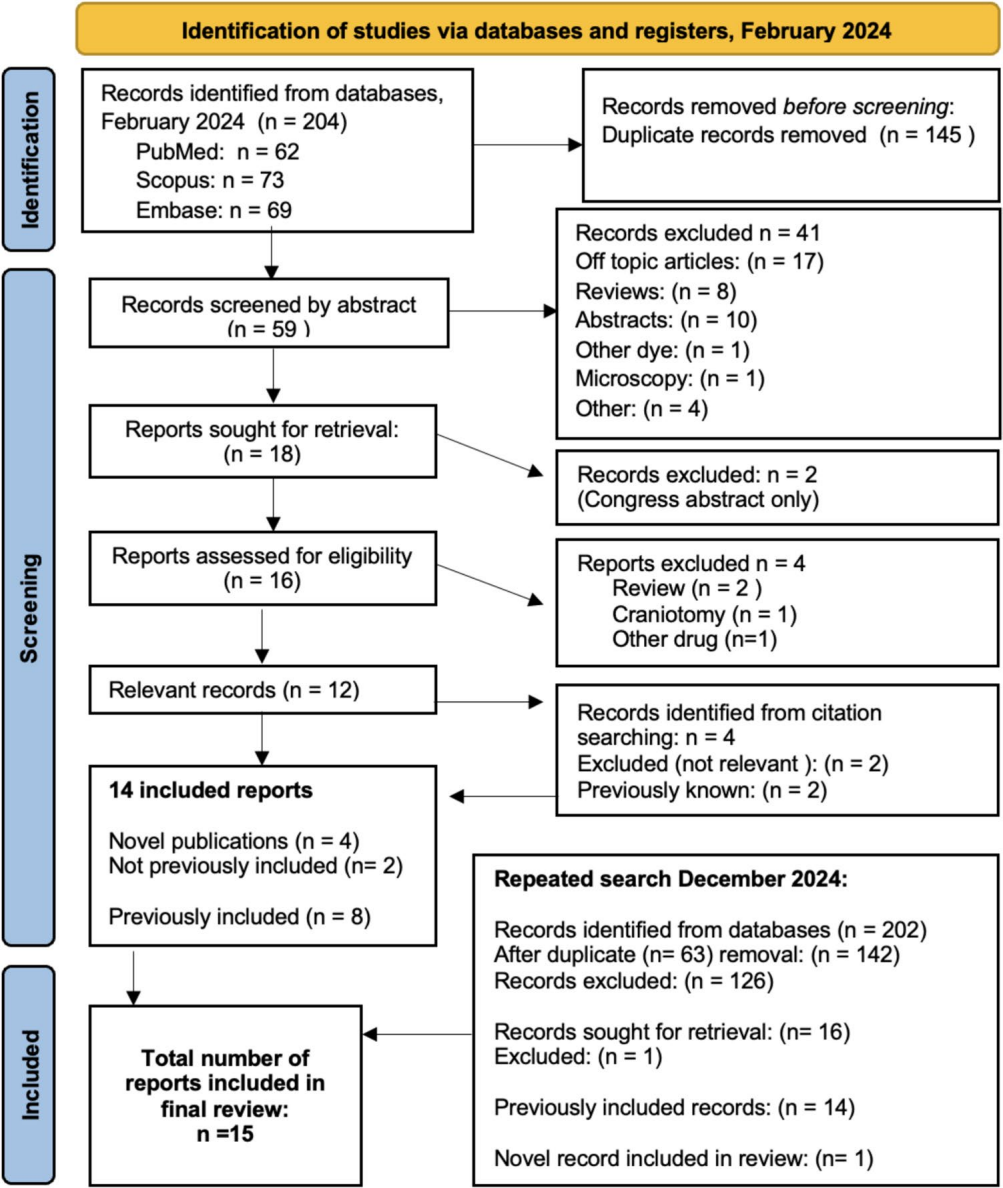
Results from the search conducted in February 2024, including the updated supplementary search from December 2024

Citation searches were performed using reference lists from included publications as well as the “citated by”- feature in PubMed (Fig. 1). Studies were initially screened based on abstract and selected full text articles were retrieved and reviewed. In cases of doubt or disagreement between reviewers, the senior author (MW) was consulted regarding inclusion. Search results were compiled and managed using EndNote X9 citation manager.

## Results

### Search results

The search in February 2024 yielded 59 results, of which 41 were excluded during the initial selection (Fig. 1). A total of 18 reports were assessed in full text for eligibility. Among these, two were found to be reviews, one utilized a different surgical method (craniotomy), and one employed a different fluorescent dye. Twelve studies met inclusion criteria [1, 5, 6, 12, 14–16, 20, 22, 27, 33, 37]. Two additional studies were added by citation-searching, one of which was a video case presentation [2, 3]. An updated search in December 2024 identified two additional potentially relevant recent publications [9, 13]. One was excluded after full text assessment, as it assessed endoscopic ICG for angiographic purposes [13]—albeit noting positive tumor fluorescence in a single case. In total, 15 publications were included in the final review (Fig. 1).

### Characteristics of included studies

The 15 studies include 264 patients with PitNET undergoing ETSS (Table 1). The total number of patients with PitNET ranged from one [2] to 39 patients [5] per study. Overall, 193 patients from the 264 were confirmed to have undergone ETSS using ICG fluorescence. Fluorescence assessment of both tumor and pituitary gland was confirmed in 141 patients (Table 1). Distribution of age, gender, tumor type and tumor size varied. Endocrinological outcomes were heterogeneously reported across studies (Table 2).

**Table 1:** Baseline data of included studies

Study	Year	Journal	PitNET (n)	ETSS w/ ICG, in Pts w/ PitNET	ICG-FL assessed in tumor and PG	ICG-dosage	ICG manufacturer / Dissolved in	Endoscope (including diameter in mm)
Studies mainly focusing on PG vs tumor-FL								
Litvack et al. [22] (Pilot)	2012	J Neurosurg	16	12	9	12.5 / 25 mg × 3	Akorn pharm / Sterile water	Storz (custom)
Verstegen et al. [37]	2016	Oper Neurosurg	10	10	9	5 mg × 1 (*n* = 1), 2 (*n* = 8) or 3 (*n* = 1)	NS / NS	Storz, 5.4 mm
Amano et al. [1]	2019	Acta Neurochir	15	15	15	6.25 / 12.5 mg x 1(n:6), 2(n:6), 3 (n:2)	Daiichi-Sankyo / NS	Storz, 5.8 mm
Inoue et al. [14]	2021	Neurosurgical focus	24	24	24	12.5 mg × 2	Daiichi- Sankyo / Sterile water	Storz, 5.8 mm
Shahein et.al [33]	2021	J Neurosurg	10	10	8	12.5 / 25 mg × 2	NS / Saline	Storz, 5.8 mm
Lee et al. [20]	2022	J Clin Neurosci	8	8	6	12.5 mg	NS / “Aqueous solution”	Stryker 1588 AIM
Muto et al. [27]	2023	World Neurosurg	25	25	25	12.5 mg	Daiichi-Sankyo / 0.9% Saline	Olympus CLV-S200-IR
Studies with other primary endpoints than assessing difference in PG vs PitNET-FL								
Hide et al. [12]	2015	J Neurosurg	26	26	4	12.5 mg × 2	Daiichi Sankyo / Sterile water	Storz, 5.8 mm
Inoue et al. [15]	2015	Int J Endocrinol	27	NS	PG-FL NS	12.5 mg (x –)	Daiichi-Sankyo / Sterile water	Storz, 5.8 mm
Catapano et al. [3]	2017	Neurosurg Review	6	6	PG-FL NS	12.5 × 2 / 25 mg × 1	NS / NS	Storz, 5.8 mm
de Notaris et al. [6]	2022	World Neurosurg	15	15	PG-FL NS	12.5 mg + 12 mg	NS / Sterile water	Storz, 5.8 mm
Berardinelli et al. [2]	2023	World Neurosurg	1	1	PG-FL NS	25 mg	NS / NS	Storz, 5.8 mm
Felbabic et al. [9]	2024	Diagnostics	34	17	17	12.5 mg × 2	Serb pharm / Sterile water	Storz 1.gen (unspecified)
SWIG-Studies								
Jeon et al. [16]	2018	Oper Neurosurg	8	8	8	5 mg/kg X 1 (16–30 h preop)	Akorn Pharm / NS	Visionsense Iridium, 4 mm
Cho et al. [5]	2018	J Neurosurg	39	16	16	5 mg/ kg × 1 (24 h preop)	Patheon* / Sterile water*	Visionsense Iridium, 4 mm
Total			**264**	**193**	**141**			

*pts* Patients, *PG* Pituitary gland, *ETSS* Endoscopic transsphenoidal surgery, *ICG* Indocyanine green, *FL* Fluorescence, *NS* Not specified in material

*Missing from manuscript, specifications according to registered trial no NCT03262636, clinicaltrials.gov

**Table 2:** Reported patient and tumor characteristics

Publication	Age*	Gender (M/F)	Tumor type distribution	Tumor size**	Tumor functionality / histology	GTR-Rate (MRI-verified)	Reported endocrine outcome (postop)
Litvack et al. [22] Safety and feasibility Pilot-study	Range 28–65	M6/ F3	**9 PitNET** (Incl.1 apoplexy)	7 macro 2 micro	4 NFPA (null-cell) 1 ACTH, 2 GH, 1 PRL 1 GH + PRL	NS	NS
Verstegen et al. [37] Clinical trial	Median 50 Range 28–74	M4/F6	**10 PitNET**	4 macro 2 micro, 4 NS	4 NFPA, 6FPA 4 ACTH, 1 GH, 1 PRL	1 not performed, 6 “normal”, 3 w/ remnant”	No hypersecretion post.op, 2 deficiencies improved, 2 remaining post op deficiencies
Amano et al. [1] Consecutive mixed case series, single center	Range 32–73	M6/ F9	**15 PitNET** 5 other	NS	6 NFPA, 9 FPA (GH): *2 null-cell, 1 ACTH, 6 GH, 2 FSH* + *LH, 3 GH* + *PRL, 1 GH* + *TSH*	NS	NS
Inoue et al. [14] 2021 Selected cases	Median 57,6 Range 24–80	M14/ F10	**24 PitNET**	20 macro (10 > 20 mm) 4 micro	18 NFPA, 6 FPA 4 GH, 1 ACTH, 1 PRL	NFPA: GTR in 16 pts (80%), 2 STR, 2 partial resection. FPA: GTR in 100%	Unchanged hormone secretion in 17/18 NFPAs. Worsened endo outcome in 1 pt. Normalization of hormone secretion in all 6 FPAs
Shahein et.al [33] Prospective cohort study	Range 25–70	M3 / F7	**10 PitNET** 10 other	8 macro 2 micro	1 NFPA (null-cell) 4 ACTH, 3 GH, 2 GH/ FSH, *1 carcinoma*	NS	NS
Lee et.al. [20] Consecutive cases	Range 28–78	M5/ F3	**8 PitNET** 2 other	7 macro 1 micro	5 NFPA, 1FPA (GH), 2 pituitary apoplexy (null-cell, GH)	7 confirmed GTR (87,5%), 1 STR	Normal post op endocrinology in 7/8 1 GH pt normalized post.op. 2 pituitary apoplexy w/ preop panhypopituitarism, did not recover post.op
Muto et al. [27] Case series	*N* = 8 Range 25–78	*N* = 8 M5 /F3	**25 PitNET**	2 macro 23 NS	20 NFPA, 5 FPA (3 GH, 2 PRL)	NIR-and MRI- assessed GTR in 22 pts, 3 STR	Hormonal disorders improved in 2 pts (1 PRL, 1 GH). Transient post.op DI in 5
Hide et al [12] Retrospective mixed case series	*N* = 38 Mean 54,5 Range 11–84	*N* = 38 M19 / F19	**26 PitNET** 12 other	NS	NS	NS	NS
Inoue et al. [15] Consecutive case series	*N* = 35 Median 55,5 Range 16–84	*N* = 35 M18/ F17	**27 PitNET** 8 other	27 macro	22 NFPA, 5 FPA (4 GH, 1 PRL)	“No or little evidence of residual tissue in all cases”	3/4 GH normalized (75%) 2 preop deficiencies w/ no change postop. 1 post op hormone replacement- not specified if **PitNET**. Rathke’s and Craniopharyngioma (*n* = 5): 1 Preop deficiency worsened post.op
Catapano et al. [3] Consecutive case series	NS	NS	**6 PitNET** 8 other	6 macro	4 NFPA, 2FPA: 2 ACTH	NS	2 pts w/ transient DI in ETSS-group, pathology NS
de Notaris et al. [6] Retrospective, selected cases	Range 18–84	M12 / F3	**15 PitNET**	14 macro 1 micro	13 NFPA, 2FPA: 1 ACTH, 1 PRL	GTR in 12 cases (80%) 3 STR	4 recoveries (2 FPA), 9 unchanged, 1 transient DI, 1 not specified. No new postop hypopituitarism
Berardinelli et al. [2] Video case	*N* = 1 49	F1	**1 PitNET**	NS	1 NFPA	GTR	“The patient experienced resolution of her symptoms”
Felbabic et al. [9] Prospective randomized study	*N* = 34 60,9 ± 11,2 ICG, *n* = 17: 62,7 ± 9,9	*N* = 34 M23/ F11 ICG: M11/F6	**34 PitNET** ICG-Leg: 17	34 macro	34 NFPA	NS	Reports on 21 cases w/ specified endocrinological complications
Jeon et al. [16] Clinical trial (SWIG)	Range 40–69 Mean 54	M4/ F4	**8 PitNET** 7 other	7 macro 1 micro	5 NFPA, 3 FPA: 3 null-cell, 2 ACTH, 3 GH	NS	NS
Cho et al. [5] Clinical trial, NCT03262636 (SWIG)	SWIG (*n* = 16) Mean 57,4 ± 11,0 OTL38 (*n* = 23) Mean 54,0 ± 17,5	M21/ F18 SWIG: M9/F7	**All cases: 39 PitNET** **SWIG- leg: 16 PitNET**	36 macro 3 micro ICG-leg: 14 macro 2 micro	21 NFPA, 13 FPA: ICG-leg: 7 NFPA, 9 FPA: 2 ACTH, 4 GH, 2 PRL, 1 TSH	All cases, *N* = 39, 20 verified GTR SWIG-leg: 5 GTR, 2 questionable, 9 incomplete	NS

*Pts* Patients, *FPA* Functional pituitary adenoma, *NFPA* Non-functional pituitary adenoma, *PRL* Prolactin, *GH* Growth Hormone, *ACTH* Adreno corticotropic hormone, *FSH* Follicle stimulating hormone, *TSH* Thyroid stimulating hormone, *ICG* Indocyanine green, *SWIG* Second window ICG, *NS* Not specified in material, *w/* With

*Reported age-distribution in years, **Microadenoma; < 10 mm, Macroadenoma ≥ 10 mm

### ICG administration

Thirteen studies [1–3, 6, 9, 12, 14, 15, 20, 22, 27, 33, 37] employed intraoperative administration of low-dose ICG. Dosage varied from 5 to 25 mg pr bolus (Table 1). ICG was acquired from various manufacturers and different solvents were used during administration (Table 1). Two studies employed an alternative protocol involving a high-dose (5 mg/kg) intravenous infusion of ICG the day prior to the procedure, referred to as "Second Window ICG" (SWIG) [5, 16].

### Endoscopes

Nine studies utilized a first-generation 5.8 mm straight rigid ICG endoscope manufactured by Karl Storz, requiring a footswitch to toggle between NIR- and white light modes (Table 1). Felbabic et al. employed an unspecified rigid ICG endoscope from Storz [9].In the low-dose ICG group, two studies reported using “second generation” endoscopes, featuring overlay-capabilities [20, 27]. The two publications reporting on the SWIG method employed a camera system from Visionsense® that utilized dual optics for simultaneous NIR/White light imaging [5, 16].

### Intraoperative pituitary gland and tumor fluorescence

Nine studies did to some extent assess both tumor and pituitary gland fluorescence after intraoperative administration of low-dose ICG [1, 9, 12, 14, 20, 22, 27, 33, 37]. Including the two studies employing the SWIG-protocol [5, 16], nine studies assessed the presence of a fluorescent gland-tumor interface during surgery as one of their main objectives. Six studies employed post hoc quantitative methods for fluorescence assessment, using imaging software tools (ImageJ® [5, 16, 27, 33, 37] and Photoshop® [12]).

Three studies, all of which utilized endoscopes with NIR/ICG overlay capabilities (Table 1), found positive tumor fluorescence in 30 cases [5, 16, 20]. Cho et al. [5] compared two different fluorescent markers (OTL-38 and SWIG), and found that all tumors in the SWIG-leg demonstrated positive intraoperative fluorescence. Five studies observed tumor tissue to be less fluorescent than the gland itself [6, 9, 22, 33, 37].

### Temporal differences

Five studies specifically assessed differences in ICG fluorescence over time, within variable timeframes. Amano et.al [1] reported that tumor fluoresence was best visualized approximately 7 min after injection, but with a shift in tissue fluorescence enhancing the gland around the 8th minute. Muto et al. described similar features. However, starting at 15 min and onward, the pituitary gland was more fluorescent than the tumor, remaining for up to 180 min [27].

Three studies focused on temporal differences in fluorescence during the “hyperacute” phase [12, 14, 15]. The speed of injection, individual hemodynamics [12] and tumor size [14] were seen to influence timing from injection to fluorescence in such early phases. Inoue et.al (2020) [14] examined the presence of gland to tumor interface and found fluorescence in the pituitary gland 22–36 s after ICG administration, and tumor fluorescence after 55–59 s. This temporal variation varied with tumor size. In their 2015 study, the same group found fluorescent signal in the pituitary gland 30–40 s after ICG injection, following tumor resection [15]. Hide et al. [12] recorded mean time at peak color value be shorter for the adenoma (30.8 s) than in the pituitary gland (33.6 s).

### Complications

Complications were reported in a heterogenous way across studies (Table 3). In summary, no study reported any complications directly related to the administration of ICG, including allergic reactions. None of the studies indicated adverse events otherwise associated with the use of ICG or the ICG-endoscope. In both studies employing the SWIG protocol [5, 16], patients received a dose of IV antihistamine when the ICG infusion was administered. In their prospective randomized study comparing 17 endoscopic ICG-cases and 17 controls, Felbabic et al. [9] found no statistically significant differences in endocrinological outcome nor complications between the groups.

**Table 3:** Reported complications

Publication	Challenges / Complications
Litvack et al. [22] 2012	No complications caused by ICG of NIR-fluorescence, nor surgical complications ICG added 15–20 min to operating time
Verstegen et al. [37] 2016	No adverse reactions to ICG 1 case had CSF-leakage 1 case had intraoperative venous bleeding
Amano et al. [1] 2019	No complications “during or after surgery that can be attributed to the ICG injection nor other general complications related to the surgery itself.”
Inoue et al. [14] 2021	All 24 patients underwent surgery without serious complications 1 case of post.op intratumoral hemorrhage from residual tumor required cerebral ventricular drainage 1 case required post.op hormone-replacement therapy w/hydrocortisone
Shahein et al. [33] 2021	“No adverse effects related to the drug” (6 month follow up)
Lee et al. [20] 2022	2 Adenoma-pts required lumbar drain due to “minimal CSF leakage” for 3 days No other complications reported
Muto et al. [27] 2023	5 cases (20%) exhibited transient diabetes insipidus (DI) in the perioperative period No pituitary dysfunction (incl permanent DI) was observed during the follow-up period
Hide et al. [12] 2015	Did not address the presence nor absence of adverse events/complications
Inoue et al. [15] 2015	“Surgery was performed in all 35 patients without serious complications.” 1 pt suffered cerebrospinal rhinorrhea that required repair surgery, 1 pt experienced decreased visual function, 1 pt required hormone replacement therapy
Catapano et al. [3] 2017	“Adverse events due to the use of the dedicated ICG endoscope and/or ICG administration did not occur.” No intraoperative surgical complications reported Post-operative complication in 2 cases (endonasal group): transient diabetes insipidus
de Notaris et al. [6] 2022	1 case of transient diabetes insipidus in the early postoperative period “No case of new postoperative hypopituitarism was noted”. “Dye cross allergy or ICG-related complications were not reported”
Berardinelli et al. [2] 2023	“At follow-up, the patient experienced resolution of her symptoms, without residues or relapses on control magnetic resonance”
Felbabic et al. [9] 2024	State a 100% incidence of immediate post-op hypopituitarism, all cases received temporary substitution with corticosteroids in the immediate post.op period 7 cases (20.6%) of early diabetes insipidus (3 in the ICG-leg, 4 in the non-ICG leg) 5 cases of permanent DI (1 in ICG-leg, 4 in non-ICG leg) 3 cases (8.8%) of temporary SIADH (2 in ICG-group, 1 in control) 6 cases of new-onset postoperative hypopituitarism 3 from ICG group, 3 from control-group Did not specifically address presence of surgical complications or complications related to ICG
Jeon et al. [16] 2018	“No immediate complications from ICG administration. All patients tolerated the high dose of ICG without any adverse events.”
Cho et al. [5] 2018	“All patients tolerated ICG or OTL38 infusions without adverse events. No permanent surgical complications were identified”

### Technical issues

Shahein et al. [33] presented two cases where ICG was not feasible and the gland could not be differentiated; one was histopathologically confirmed to be a carcinoma, and the other case was a repeat-surgery following primary surgery a year prior. Lee and Lee [20] described two cases with pituitary apoplexy where ICG was deemed “not useful”. DeNotaris et al. [6] noted that in their population, where 6 out of 15 patients had undergone previous surgeries, optimal visualization of the pituitary gland was achieved in all but one case, where the patient had surgery a year earlier. In their pilot-study, Litvack et al. [22] originally enrolled 16 patients of which 12 underwent surgery with ICG. In addition, a technical failure occurred in the first three cases, leading to study protocol revisions and dose escalation over the first 8 cases. They found that ICG added 15 to 20 min to the operative time. Due to intraoperative bleeding, Verstegen et al. [37] were unable to interpret ICG signal from the pituitary gland and tumor in one case. This was supported by Catapano et al. [3], who stated that it *“was mandatory to ensure a clear and bloodless operative field before using ICG to avoid false positive fluorescence visualization.”* Amano et al. [1] described that tumor fluorescence intensity depended significantly on the distance between the fluorescent object and endoscope lens.

### Data analysis

Systematic reviews commonly incorporate meta-analyses and risk of bias assessments to synthesize evidence and evaluate study reliability. These methods are not feasible in the present study due to the nature of included literature. We mainly identified case reports and case series, which inherently lack control groups and standardized outcome measures.

## Discussion

Differentiating normal pituitary gland from tumor tissue remains a challenge in endoscopic pituitary surgery. Accurate identification of tumor margins is critical for achieving optimal surgical outcomes, including complete tumor resection while preserving normal gland function. The morphological and functional overlap between normal glandular tissue and tumor tissue complicates this process, requiring advanced imaging techniques, intraoperative tools, and histological confirmation to enhance surgical precision. Over a decade ago, Litvack et al. [22] hypothesized that ICG could be used to visualize differences in vascular density between pituitary tumor and surrounding structures, by using ICG guided fluorescence in endoscopic transsphenoidal surgery. They found that the pituitary gland had an increased ICG signal compared to the tumor. Despite a multitude of variabilities in protocols, this finding has been supported by most studies included in this review [1, 2, 6, 9, 22, 27, 33, 37]. This corresponds well with the general idea that PitNET are less vascularized than the pituitary gland itself. However, the microvascular architecture and complexity of PitNET may vary, which could lead to different enhancement patterns and thus at least partially explain the heterogenous findings in some studies [7, 35].

### Temporal aspects

As tumor resection requires time and the identification of a tumor to pituitary gland interface may become more evident in later phases of surgery, the temporal dynamics of ICG are important to understand. Temporal changes in fluorescence dynamics, causing a shift between adenoma and pituitary gland fluorescence within the first 15 min after ICG-administration has been reported in several cases [1, 27]. Some included studies found a single dose of ICG to be sufficient to maintain pituitary gland fluorescence throughout the entire procedure [3, 6, 33]. Although this is not ubiquitously reported [37], these findings emphasize the potential of continuous low-dose ICG fluorescent guidance throughout the resection phase.

### Correlation between preoperative MRI, histopathology, and intraoperative fluorescence

Few studies have systematically assessed intraoperative observations using histopathological assessment [5, 16, 27]. All report a high sensitivity, but varying specificity, indicating that ICG fluorescence in tumor tissue carries a risk of being unspecific, and interpretation of intraoperative fluorescence should be done with care. A correlation between preoperative Gd-MRI contrast enhancement and ICG-fluorescence, whether in tumor or pituitary gland, has also been observed [16, 27, 33]. As with intraoperative ICG-fluorescence, a difference in tumor microvasculature could also explain a difference in preoperative Gd-contrast enhancement.

### ICG properties

There are large variations in administration protocols and dosage of ICG among included studies, and determining their significance in terms of fluorescence outcome is challenging.

Another potential source of bias among included studies, is that different ICG products and formulations have been employed (Table 1). There is a variety of different ICG products as well as different solvents used across studies, that have not been assessed systematically, which potentially carry a risk of differing dynamics in ICG tissue distribution.

An ex-vivo study examining the absorption spectrum and osmolarity of three different ICG products found a difference in absorption spectrum depending on both solvent and concentration of ICG [11]. Thus, small differences in pharmaceutical formulations and solvent could theoretically carry the potential to affect intraoperative fluorescence detection.

Some studies report on using sterile water to dilute ICG, whereas others report utilizing 0.9% sodium chloride. In addition, there are differences in ICG formulations between manufacturers. For example, ICG delivered from Serb Pharmaceuticals (Infracyanine®) does not contain iodine (contrary to IC-Green from Akorn and Patheons ICG formulations) and utilize 5% glucose as a solvent medium. Complexity further increases with different endoscope NIR-range settings. Differences in formulation and solvent, together with NIR-range settings left alone, could in theory cause the ICG fluorescence spectrum being dose and application dependent in vivo. Generally, future studies should therefore also focus on different formulations and dosages of ICG used, as well as the differences in detection.

### Technological considerations

Advancements in fluorescence-guided endoscopic transsphenoidal surgery have been lagging, compared to other fluorescence-guided neurosurgical fields. This discrepancy is foremost due to the lack of fluorescence-compatible endoscopes suitable for endonasal transsphenoidal approaches. Most endoscopic procedures and intraoperative fluorescence assessments presented in this review were performed using first-generation endoscopes measuring 5.8 mm in diameter. These endoscopes require toggling between NIR and white light modes, which may impede adequate intraoperative assessment of fluorescence duration and fading. The delicate structures in and around the sella also prohibit tumor resection under pure fluorescence mode, providing a blurry dark anatomical picture with fluorescence signal. Although most of the authors of the included studies found endoscope integrated ICG to be a promising tool, they underline the need for technical improvements in terms of a smaller diameter, overlay white light imaging or angled endoscopes [1, 3, 6, 9, 12, 33, 37]. It is notable that studies that have described durable positive tumor fluorescence all employed endoscopes with overlay capabilities during surgery [5, 16, 20].

Recently, second-generation neurosurgical fluorescence compatible endoscopes have been introduced, offering real-time overlay images that combine white light and fluorescence modes during tumor dissection [13, 20]. This innovation allows for precise microsurgical tumor dissection under white light conditions, augmented by a continuous fluorescence signal overlay. However, a significant barrier to acquiring these advanced neurosurgical endoscopic optics in many European neurosurgical departments is noteworthy and stems from the European Union (EU) Medical Device Regulation (MDR; (EU) 2017/745) which came into effect in May 2021. Due to bureaucratic challenges state-of-the art neuro-endoscopic optics are still not readily available in the EU.

### Interpretation of intra-operative fluorescence signal

The interpretation of intraoperative fluorescence intensity varies based on the surgeon’s experience, as ICG is not a tumor-specific marker. Fluorescence should therefore be considered an adjunct to preoperative imaging, white light visualization, and tactile assessment of tissue consistency. This subjectivity makes fluorescence interpretation challenging to quantify. While practical and interpretable fluorescence guidance is ideal, discrepancies between studies arise from both technical variations and differing interpretations. Some studies have employed post-hoc quantitative methods to improve objectivity, but these often lack histopathological validation and corresponding intraoperative tissue identification. Although post-hoc quantitative analysis can enhance research insights, it provides limited real-time value for surgeons. Signal intensity and persistence depend on several factors, such as tissue properties, ICG dosage, endoscopic device sensitivity, light source distance, and tissue manipulation- which may temporarily increase perfusion and thus fluorescence.

### Challenges and limitations

There is currently no consensus on the optimal use of ICG fluorescence in ETSS for PitNET. While small studies suggest potential benefits, significant variability in study design, ICG administration protocols, fluorescence assessment methods, and endoscopic equipment contributes to heterogeneous and often conflicting results. The available evidence is mainly limited to small case series, making it prone to reporting bias and selective publication of positive findings. The included studies vary in ICG dosage, timing, and imaging techniques, preventing direct comparisons and consistent conclusions. Some employ quantitative fluorescence analysis (e.g., ImageJ® or Photoshop®-based post-hoc assessments), while others rely on subjective intraoperative observations, leading to inconsistent reporting and interpretation. The absence of controlled trials makes it impossible to determine causality between ICG fluorescence and improved surgical outcomes, such as higher gross total resection (GTR) rates or better endocrinological outcomes. Additionally, traditional meta-analytic techniques (e.g., I^2^ heterogeneity measurements) require methodological uniformity, which case series inherently lack. Applying such statistical methods would not yield reliable or clinically meaningful conclusions. Also, potential financial conflicts of interest are noted in studies involving “second-generation” equipment [5, 16, 27]. Studies reporting positive fluorescence findings are more likely to be published, potentially overestimating the clinical utility of ICG fluorescence. As a result, current data are insufficient to determine whether intraoperative ICG can improve gross total resection rates, endocrinological outcomes, nor reduce recurrence or complication rates.

## Conclusions

This systematic review evaluates the current literature on intraoperative ICG fluorescence during ETSS for PitNET. The 15 included studies demonstrate significant heterogeneity in methodology, ICG administration, imaging systems, and fluorescence interpretation, making direct comparisons challenging. Despite these variations, the collective findings provide valuable insights into the potential applications, limitations, and technical challenges of ICG in pituitary surgery.

While ICG fluorescence shows promise as a real-time angiographic tool and a potential marker for pituitary tissue differentiation, there remains no clear consensus on its efficacy as an intraoperative tumor marker. The absence of standardized protocols, objective outcome measures, and controlled comparative studies limits its widespread adoption. Additionally, variability in fluorescence timing, ICG dosing strategies, and endoscopic technology further complicates interpretation and reproducibility across clinical settings.

Future research should prioritize well-designed, prospective, studies with standardized fluorescence assessment protocols to determine whether ICG fluorescence can improve tumor resection rates, enhance pituitary gland preservation, and ultimately influence patient outcomes. ICG fluorescence remains an adjunctive intraoperative tool with potential benefits, but further validation is required to establish its definitive role in pituitary surgery.

## Supplementary Information

Below is the link to the electronic supplementary material.

Supplementary file1 (DOCX 16 KB)

## Data Availability

No datasets were generated or analysed during the current study.
