# Supplementary material for: Indocyanine green fluorescence in endoscopic transsphenoidal resection of pituitary neuroendocrine tumors: a systematic review
Source: Acta Neurochir (Wien). 2025 Mar 28;167(1):92. doi: 10.1007/s00701-025-06500-z (PMC11953215; doi:10.1007/s00701-025-06500-z)
Supplement: Supplementary file 1 — Supplementary file1 (DOCX 16 KB) [file 701_2025_6500_MOESM1_ESM.docx]

**Supplementary table 1:** search terms used for each database:

| **PubMed** |  |
| --- | --- |
| (Pituitary adenoma) AND (icg) | **(pituitary adenoma) AND (icg)**  ("pituitary neoplasms"[MeSH Terms] OR ("pituitary"[All Fields] AND "neoplasms"[All Fields]) OR "pituitary neoplasms"[All Fields] OR ("pituitary"[All Fields] AND "adenoma"[All Fields]) OR "pituitary adenoma"[All Fields]) AND "icg"[All Fields]  **Translations**  **pituitary adenoma:** "pituitary neoplasms"[MeSH Terms] OR ("pituitary"[All Fields] AND "neoplasms"[All Fields]) OR "pituitary neoplasms"[All Fields] OR ("pituitary"[All Fields] AND "adenoma"[All Fields]) OR "pituitary adenoma"[All Fields] |
| (Pituitary adenoma) AND (indocyanine green) | **(pituitary adenoma) AND (indocyanine green)**  ("pituitary neoplasms"[MeSH Terms] OR ("pituitary"[All Fields] AND "neoplasms"[All Fields]) OR "pituitary neoplasms"[All Fields] OR ("pituitary"[All Fields] AND "adenoma"[All Fields]) OR "pituitary adenoma"[All Fields]) AND ("indocyanine green"[MeSH Terms] OR ("indocyanine"[All Fields] AND "green"[All Fields]) OR "indocyanine green"[All Fields])  **Translations**  **pituitary adenoma:** "pituitary neoplasms"[MeSH Terms] OR ("pituitary"[All Fields] AND "neoplasms"[All Fields]) OR "pituitary neoplasms"[All Fields] OR ("pituitary"[All Fields] AND "adenoma"[All Fields]) OR "pituitary adenoma"[All Fields]  **indocyanine green:** "indocyanine green"[MeSH Terms] OR ("indocyanine"[All Fields] AND "green"[All Fields]) OR "indocyanine green"[All Fields] |
| (((pituitary adenoma[MeSH Terms]) AND (Pituitary adenoma))AND ((indocyanine green[MeSH Terms]) AND (Indocyanine green))) | "pituitary neoplasms"[MeSH Terms] AND ("pituitary neoplasms"[MeSH Terms] OR ("pituitary"[All Fields] AND "neoplasms"[All Fields]) OR "pituitary neoplasms"[All Fields] OR ("pituitary"[All Fields] AND "adenoma"[All Fields]) OR "pituitary adenoma"[All Fields]) AND ("indocyanine green"[MeSH Terms] AND ("indocyanine green"[MeSH Terms] OR ("indocyanine"[All Fields] AND "green"[All Fields]) OR "indocyanine green"[All Fields]))  **Translations**  **pituitary adenoma[MeSH Terms]:** "pituitary neoplasms"[MeSH Terms]  **Pituitary adenoma:** "pituitary neoplasms"[MeSH Terms] OR ("pituitary"[All Fields] AND "neoplasms"[All Fields]) OR "pituitary neoplasms"[All Fields] OR ("pituitary"[All Fields] AND "adenoma"[All Fields]) OR "pituitary adenoma"[All Fields]  **indocyanine green[MeSH Terms]:** "indocyanine green"[MeSH Terms]  **Indocyanine green:** "indocyanine green"[MeSH Terms] OR ("indocyanine"[All Fields] AND "green"[All Fields]) OR "indocyanine green"[All Fields] |
| (Pituitary adenoma) AND (ICG) AND (transsphenoidal) | ("pituitary neoplasms"[MeSH Terms] OR ("pituitary"[All Fields] AND "neoplasms"[All Fields]) OR "pituitary neoplasms"[All Fields] OR ("pituitary"[All Fields] AND "adenoma"[All Fields]) OR "pituitary adenoma"[All Fields]) AND "ICG"[All Fields] AND ("transsphenoid"[All Fields] OR "transsphenoidal"[All Fields] OR "transsphenoidally"[All Fields])  **Translations**  **Pituitary adenoma:** "pituitary neoplasms"[MeSH Terms] OR ("pituitary"[All Fields] AND "neoplasms"[All Fields]) OR "pituitary neoplasms"[All Fields] OR ("pituitary"[All Fields] AND "adenoma"[All Fields]) OR "pituitary adenoma"[All Fields]  **transsphenoidal:** "transsphenoid"[All Fields] OR "transsphenoidal"[All Fields] OR "transsphenoidally"[All Fields] |
| **Scopus** |  |
| Searched:  «Pituitary adenoma» and «ICG” or “Indocyanine green” | TITLE+ABS+KEY; “Pituitary adenoma” and “icg”  TITLE+ABS+KEY; “Pituitary AND adenoma AND indocyanine AND green”  TITLE+ABS+KEY; “Pituitary AND adenoma AND indocyanine AND green AND Transsphenoidal”  *TITLE+ABS+KEY; “Pituitary AND adenoma AND icg AND transsphenoidal”*  ( ALL ( pituitary AND adenoma ) AND ALL ( indocyanine AND green AND icg ) ) |
| **Embase** |  |
| Embase Classic+Embase 1947 to 2024 | “exp hypophysis adenoma/ and exp indocyanine green/ “ “exp pituitary adenoma/ and exp indocyanine green/ “  “Pituitary adenoma OR hypophysis adenoma AND icg OR indocyanine green”  “Pituitary adenoma or hypophysis adenoma AND icg OR indocyanine green AND transsphenoidal surgery” |
| **Medline** |  |
|  | exp pituitary adenoma/ and exp indocyanine green/ (15) |
